# Management of a Transaxial, Tricompartmental Gunshot Injury in a Low-Resource Tertiary-Care Center in the Anglosphere Caribbean

**DOI:** 10.7759/cureus.47516

**Published:** 2023-10-23

**Authors:** Jaime T Lee Young, Johnathan K Jarvis

**Affiliations:** 1 General Surgery, Port of Spain General Hospital, Port of Spain, TTO

**Keywords:** gun shot wounds, low resource center, thoracoabdominal injury, transaxial penetrating injury, penetrating thoracic injury, penetrating abdominal injury, trauma

## Abstract

Trauma, both penetrating and blunt, consists of a significant percentage of surgical admissions in Caribbean hospitals. Due to financial constraints, ideal resources for optimal surgical management are not always available. Despite these disadvantages, successful outcomes for complex, emergent cases are achieved through a combination of timely clinical assessment, intervention, and ingenuity in using the resources at hand.

In this case report, we describe a 17-year-old male who suffered major visceral injuries and presented in extremis from a single gunshot wound. While fleeing the scene of a crime, he was shot in his right pelvis, with the projectile exiting his left thorax. Injuries matching a transaxial gunshot trajectory that crossed the diaphragm and involved the pelvic, abdominal, and thoracic cavities were found on exploratory laparotomy. He survived through prompt surgical intervention and aggressive resuscitation during his postoperative intensivist care, a resource often unavailable in this setting.

The patient’s prognosis would have been guarded even in a developed country setting. This case highlights the potential that Caribbean healthcare institutes possess, and that given an improvement in resources, we can aim to match a developed country’s standard of healthcare.

## Introduction

Penetrating trauma occurs when an object pierces the skin and enters the body, creating a wound [1]. Management of penetrating trauma is theoretically forthright, with Advanced Trauma Life Support protocols playing a significant role in the initial treatment approach and resuscitation of the patient. The treatment approach may become complicated when surgical intervention is necessary, especially in gunshot wounds, where the kinetic energy involved may cause damage tangential to the missile’s trajectory, resulting in occult injuries far from the expected track. Body habitus can influence patient outcomes, with injury severity being inversely proportional to adiposity [2]. One study from a tertiary-level hospital in Tehran, Iran, revealed that 61% of patients with penetrating abdominal trauma over a one-year period required laparotomy [3].

The objective of this case report is to demonstrate that despite the challenges faced in a developing nation’s healthcare setting, a high standard of patient care can still be met in the management of multi-penetrating thoraco-abdominal trauma.

## Case presentation

A 17-year-old male patient with no known co-morbidities was involved in an attempted home invasion. The patient presented to the accident and emergency department with a systolic blood pressure of 140-150 mmHg and a pulse rate of 90-100 bpm after being shot by the homeowner. On general observation, the patient was underweight (body mass index <18.5 kg/m^2^), diaphoretic, and in mild pain. A single 1 cm^2^ circular, symmetrical wound was found in the right gluteal region, approximately 2 cm inferior and 2 cm posterior to the anterior superior iliac spine. A slightly larger, elliptical 3 cm^2^ wound with ragged skin edges was found on the anterolateral aspect of the left chest wall between ribs six and seven, just anterior to the anterior axillary line.

A cardiovascular examination of the patient was normal, and a respiratory examination revealed decreased breath sounds over the base of the left lung associated with dullness on percussion. The patient exhibited peritonitis and absent bowel sounds, but a rectal examination was normal. An extended focused assessment with sonography for trauma (eFAST) revealed a left hemothorax prompting the insertion of a thoracostomy tube, which drained 200 mL of blood, with concomitant urinary catheterization.

The results of his initial emergency department complete blood count and renal function tests are listed in Table 1. A blood sample was also collected and sent to the laboratory for group- and cross-matching.

**Table 1:** Initial laboratory investigations in the emergency department

Laboratory investigation	Result	Normal Range
Hemoglobin	12.7 g/dL	13.0- 17.0 g/dL
White blood cell count	6.9x10­^9 ^/L	4.0x10^9^ - 10.0x10^9^/L
Platelets	204x10^9^/L	150x10^9^- 410x10^9^/L
Sodium	140 mmol/L	135.0- 145.0 mmol/L
Potassium	5.6 mmol/L	3.5- 5.1 mmol/L
Blood urea nitrogen	11.6 mg/dL	6.0- 23.0 mg/dL
Creatinine	0.8 mg/dL	0.7- 1.2 mg/dL

The patient received continuous fluid resuscitation, as his blood pressure decreased to 120-130 mmHg after an initial 1 L of Ringer’s lactate solution. After receiving 3 L of intravenous crystalloids, the patient was noted to have worsening vital signs, with systolic blood pressures beginning to fluctuate between 100 and 110 mmHg, mean arterial blood pressures of 50-55 mmHg, and a heart rate of 110 bpm in conjunction with increasing abdominal pain and worsening anxiety. A preoperative X-ray was not available as the only portable X-ray machine in the facility was not functional. The patient underwent an emergency exploratory laparotomy. Injuries consistent with a transaxial gunshot trajectory that crossed the diaphragm and involved the pelvic, abdominal, and thoracic cavities were found. These included an entry wound just inferior and posterior to the anterior superior iliac spine, a lacerated cecum, several small bowel injuries, transverse colon enterotomies, posterior and anterior gastric wall lacerations, a left hemi-diaphragmatic injury, and an exit wound on the patient’s left thoracic wall between ribs six and seven, just anterior to the anterior axillary line. A full description of the internal injuries and their associated repair method are listed in Table 2.

**Table 2:** The various internal injuries in the patient, with their respective repair method

Injury	Repair
1.5cm left hemi-diaphragmatic laceration	Simple interrupted closure with 0 PDS
2cm posterior gastric wall laceration	2-layer repair: first layer- 0 PDS continuous, second layer- 0 vicryl interrupted
2cm anterior gastric wall laceration along the superior aspect of the greater curvature	2-layer repair: first layer- 0 vicryl continuous, second layer- 0 vicryl interrupted
7cm anterior gastric wall longitudinal laceration in the antrum	
<25% circumferential jejunal enterotomy 6cm from duodenojejunal flexure	2.0 vicryl primary repair
Two >50% circumferential jejunal enterotomies involving a 10cm segment of jejunum approximately 12cm from the duodenojejunal flexure	The 10cm segment of jejunum between the enterotomies was resected and an end-to-end anastomosis was created with 2-layer closure utilizing 2.0 vicryl
<25% 1cm enterotomy along proximal ileum	2-layer primary repair with 0 vicryl
Two <50% circumference enterotomies along the distal ileum	
Lacerated cecum with significant wall cecal wall loss	A right hemicolectomy was done, a side-to-side ileocolic anti-peristaltic stapled anastomosis was created with a 75 mm linear stapler, and the enterotomy was closed with 2-0 vicryl
Posterior >50% circumferential enterotomy at the midpoint of the transverse colon with associated mesenteric injury	

Hemostasis was achieved in the patient, and two 15-French Blake drains were left in situ, draining Morrison’s pouch and the pelvis. The initial thoracostomy tube was repositioned and aspirated, as it was no longer oscillating. Upon aspiration, a clot was retrieved resolving the obstruction and the underwater seal began to oscillate again. The patient was subsequently transferred to the intensive care unit, as he required norepinephrine 8 mg/50 ml at a rate of 10 ml/hour to maintain a mean arterial blood pressure above 60 mmHg. He was started on piperacillin-tazobactam 4.5 g IV TDS and was unable to be extubated, his arterial blood gases revealed persistent mixed acidosis with associated hypoxemia.

He remained intubated and on norepinephrine infusion on postoperative day 1. His vitals remained stable with a blood pressure of 121/71 mmHg, a maximum heart rate of 103 bpm, and a temperature of 36.4°C. His postoperative day 1 complete blood count is shown in Table 3 below.

**Table 3:** Postoperative day 1 complete blood count

Laboratory investigation	Result	Normal range
Hemoglobin	8.3 g/dL	13.0- 17.0 g/dL
White blood cell count	4.3x10^9^/L	4.0x10^9 ^- 10.0x10^9^/L
Platelets	170x10^9^/L	150x10^9 ^- 410x10^9^/L

On day two in the intensive care unit, the patient was extubated and no longer required inotropic support. He maintained a blood pressure of 142/82 mmHg, a maximum heart rate of 100 bpm, and a temperature of 36.8°C. His postoperative day 2 complete blood count is shown in Table 4.

**Table 4:** Postoperative day 2 complete blood count

Laboratory investigation	Result	Normal range
Hemoglobin	7.2g/dL	13.0-17.0g/dL
White blood cell count	19.5x10^9^/L	4.0x10^9^-10.0x10^9^/L
Platelets	160x10^9^/L	150x10^9^-410x10^9^/L

In response to the decrease in hemoglobin and rise in white blood cell count, a computed tomography (CT) scan of the abdomen and pelvis with intravenous contrast was performed. This revealed a dilated small bowel, no extravasation of contrast, no anastomotic leak, and no pelvic/abdominal collections. The intercostal tube and abdominal drains were removed on day 5 postoperatively. The patient was discharged to ward care on postoperative day 6, and oral intake of clear fluids was initiated.

**Figure 1:**
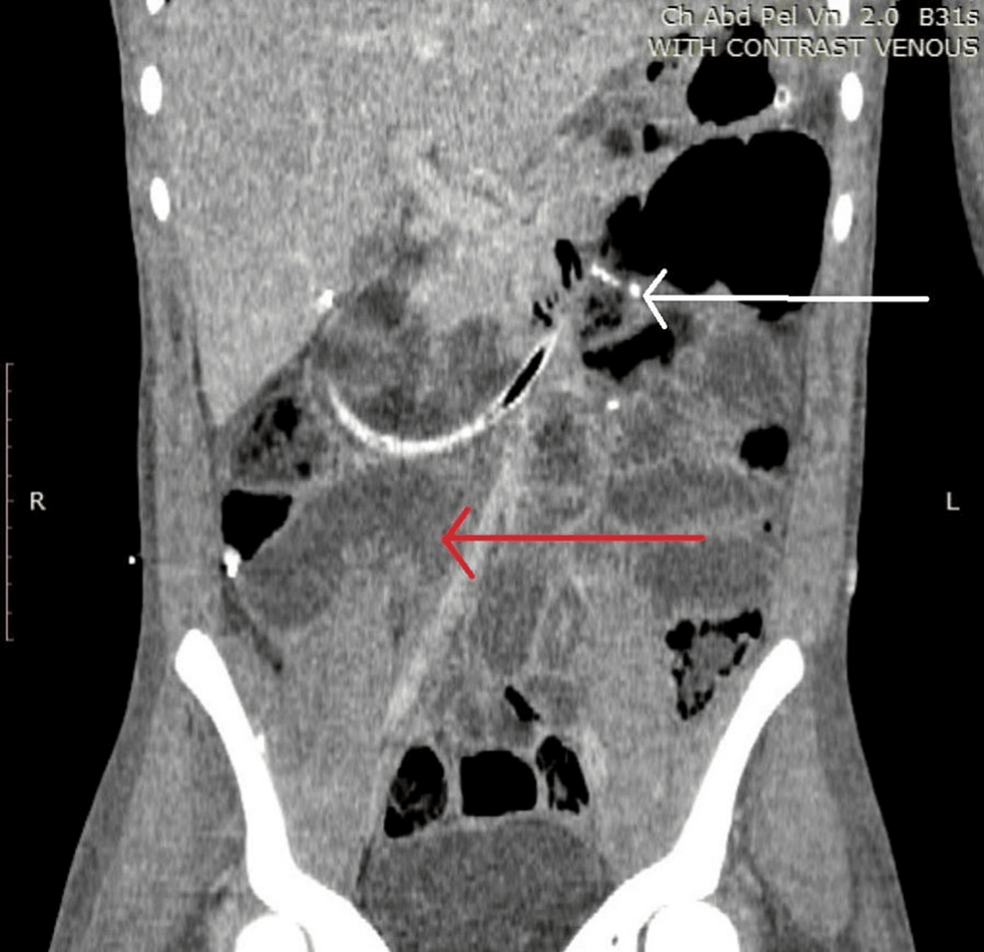
Coronal slice of the computed tomography scan with intravenous contrast in the venous phase showing dilated loops of the small bowel, with the ileocolic anastomotic staple line being seen in the left upper quadrant White arrow - ileocolic anastomotic staple line; red arrow - dilated loop of the small bowel

**Figure 2:**
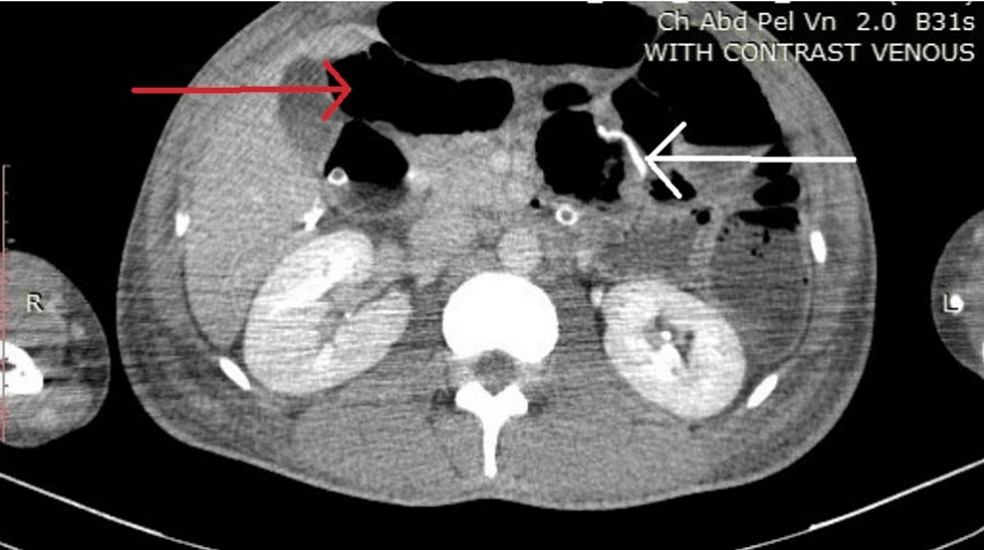
Axial slice of the computed tomography scan with intravenous contrast in the venous phase showing dilated loops of the small bowel and the suture line of the ileocolic anastomosis White arrow - ileocolic anastomotic suture line; red arrow - dilated loop of small bowel

The patient’s white blood cell count subsequently returned to normal levels (10 × 10^9^/L). His hemoglobin rose to 8.3 g/dL after receiving a transfusion of one unit of packed red blood cells. The patient was discharged from tertiary-level care on postoperative day 10, tolerating normal oral intake with full return of bowel function after conservative management of the ileus, ambulating, and having completed 10 days of intravenous antibiotics.

## Discussion

A 2019 global study on homicide by the United Nations Office on Drugs and Crime (UNODC) estimated that firearms were involved in roughly 75% of all homicides in the Americas and accounted for more than one-quarter of homicides globally that year [4]. Homicide was one of the five leading causes of death in both sexes and all races for those aged 1 to 24 years in the United States of America in 2020 [5]. Therefore, the importance of appropriate management of gunshot wounds cannot be understated.

Gunshot wounds are divided into two categories: high-velocity (large-caliber bullets) and low-velocity (small-caliber bullets) injuries. Large-caliber bullets often cause severe soft-tissue damage due to their momentum creating a cavitation effect [6]. In these cases, it is prudent to consider the patient’s body mass index. The lack of additional abdominal soft tissue (muscle and adipose) has been theorized to play a role in the severity of penetrating injuries, more classically in stab wounds compared to gunshot wounds, but the concept is similar in both cases. Missiles have more mass to traverse, which leads to increased dissipation of kinetic energy, leading to a greater deceleration force compared to a projectile that has less mass to travel through, leading to a “cushioning” effect [2].

Maximal conversion of kinetic energy to potential energy occurs as a projectile exits the body, explaining why the patient’s stomach suffered multiple injuries. In particular, the cecum sustained a significant degree of injury due to the ricocheting of the bullet against the bony pelvis. Bullet trajectory, although traditionally more involved in forensic pathology, is an important surgical consideration. One retrospective study done in 1996 revealed that a transaxial bullet path (i.e., one that traverses the truncal midline) carries a significantly elevated risk of extensive visceral damage, operative complexity, and mortality [7]. In this patient, the bullet entered the right pelvis and exited from the left hemithorax involving three compartments - the pelvis, the abdomen, and the thorax, and the trajectory matched the locations of the injuries found on surgical exploration. Thoracoabdominal injury is correlated with an increased risk of major cardiopulmonary insult, with the latter being directly linked to the poor outcomes typically seen in thoracoabdominal wounds, as exhibited by patient mortality of 31% in a four-year retrospective study at a level 1 trauma center in the United States [8].

In our case report, it can be assumed that the patient’s prognosis would have been poor even in a developed country. In a developing country, patient outcomes can also be impacted by resource unavailability. Postoperatively, the patient would have triggered the initiation of a massive transfusion protocol due to his combined hemodynamic instability and drop in hemoglobin, which would have greatly benefitted the hemodynamics of the case in other settings. The institute utilizes a 1:1:1 ratio of packed red blood cells, fresh frozen plasma, and platelets. However, he was only able to obtain one unit of packed red blood cells postoperatively due to a lack of available blood products.

Another example of resource unavailability was having to use vicryl instead of PDS for the second layer of the posterior gastric laceration, as PDS is preferred for gastric repairs. A similar situation was encountered during intestinal repairs. After the gastric repair, the ileal injuries were addressed next, as there was greater hemorrhaging from the ileum as compared to the jejunum. Once repaired, it was found that there was no more 0 vicryl available. Due to the thicker jejunal wall, 0 vicryl would have been preferred instead of the 2.0 vicryl used. This highlights the importance of resource consideration in a resource-poor institute. In addition, a postoperative CT scan was considered for the assessment of any occult injuries. Although the patient was stable by the second postoperative day, a CT scan was not obtained until day six due to transport limitations to an offsite imaging facility.

The positive outcome of the patient was due to timely operative intervention preventing further hemorrhage. The decision for immediate surgery was made when he went into Advanced Trauma Life Support Class II hemorrhagic shock despite 3 L of intravenous fluids, this also would classify him as a non-responder to initial fluid resuscitation. The absence of cardiopulmonary injury or retroperitoneal involvement also played a role, as these would have complicated the anatomy of the case, with an associated 18%-60% increase in mortality rate regarding traumatic retroperitoneal injuries [9]. The relatively young age of the patient, as compared to the elderly or one suffering from co-morbidities, would have been beneficial for his hemodynamic physiology.

## Conclusions

Despite being common, penetrating trauma exists as a spectrum of injury, varying from simple lacerations to complex surgical cases. As seen in this case report, the successful management of patients with significant thoracoabdominal injury is indeed possible in a low-resource setting. However, it should be noted that there exists a scarcity of trauma-related research within the Caribbean in terms of the techniques and outcomes of surgical cases.

This case shows the surgical potential that Caribbean nations possess, as multiple factors worked together to allow the patient to overcome the limitations present. By combining surgical skills with tenacity in pursuing the best possible patient outcome, the successful and safe discharge of high-risk trauma patients is indeed possible in a developing-world setting.
